# Insight Into Mosquito GnRH-Related Neuropeptide Receptor Specificity Revealed Through Analysis of Naturally Occurring and Synthetic Analogs of This Neuropeptide Family

**DOI:** 10.3389/fendo.2019.00742

**Published:** 2019-11-01

**Authors:** Azizia Wahedi, Gerd Gäde, Jean-Paul Paluzzi

**Affiliations:** ^1^Department of Biology, York University, Toronto, ON, Canada; ^2^Department of Biological Sciences, University of Cape Town, Cape Town, South Africa

**Keywords:** GnRH-related neuropeptides, G protein-coupled receptor, structure activity relationships, adipokinetic hormone (AKH), corazonin (CRZ), AKH/CRZ-related peptide (ACP)

## Abstract

Adipokinetic hormone (AKH), corazonin (CRZ), and the AKH/CRZ-related peptide (ACP) are neuropeptides considered homologous to the vertebrate gonadotropin-releasing hormone (GnRH). All three *Aedes aegypti* GnRH-related neuropeptide receptors have been characterized and functionally deorphanized. Individually they exhibit high specificity for their native ligands, prompting us to investigate the contribution of ligand structures in conferring receptor specificity for two of these receptors. Here, we designed a series of analogs based on the native ACP sequence and screened them using a heterologous system to identify critical residues required for ACP receptor (ACPR) activation. Analogs lacking the carboxy-terminal amidation, replacing aromatics, as well as truncated analogs were either completely inactive or had very low activities on ACPR. The polar threonine (position 3) and the blocked amino-terminal pyroglutamate are also critical, whereas ACP analogs with alanine substitutions at position 2 (valine), 5 (serine), 6 (arginine), and 7 (aspartate) were less detrimental including the substitution of charged residues. Replacing asparagine (position 9) with an alanine resulted in a 5-fold more active analog. A naturally-occurring ACP analog, with a conserved substitution in position two, was well tolerated yet displayed significantly reduced activity compared to the native mosquito ACP peptide. Chain length contributes to ligand selectivity in this system, since the endogenous octapeptide Aedae-AKH does not activate the ACPR whereas AKH decapeptides show low albeit significant activity. Similarly, we utilized this *in vitro* heterologous assay approach against an *A. aegypti* AKH receptor (AKHR-IA) testing carefully selected naturally-occurring AKH analogs from other insects to determine how substitutions of specific residues in the AKH ligand influence AKHR-IA activation. AKH analogs having single substitutions compared to Aedae-AKH revealed position 7 (either serine or asparagine) was well tolerated or had slightly improved activation whereas changes to position 6 (proline) compromised receptor activation by nearly 10-fold. Substitution of position 3 (threonine) or analogs with combinations of substitutions were quite detrimental with a significant decrease in AKHR-IA activation. Collectively, these results advance our understanding of how two GnRH-related systems in *A. aegypti* sharing the most recent evolutionary origin sustain independence of function and signaling despite their relatively high degree of ligand and receptor homology.

## Introduction

In invertebrates there exist three neuropeptide systems of which the mature peptides as well as their cognate G protein-coupled receptors (GPCRs) are structurally similar, and collectively, they are related to the vertebrate gonadotropin releasing hormone (GnRH) system and suggested to form a large peptide superfamily (1–4). These neuropeptide systems are called the adipokinetic hormone (AKH)/red pigment-concentrating hormone (RPCH) family, the corazonin (CRZ) family, and the structurally intermediate adipokinetic hormone/corazonin-related peptide (ACP) family. AKHs are primarily or exclusively produced in neurosecretory cells of the corpus cardiacum, and a major function is mobilization of energy reserves stored in the fat body, thus providing an increase of the concentration of diacylglycerols, trehalose, or proline for locomotory active phases in the haemolymph. In accordance with this function, AKH has been shown to activate the enzymes glycogen phosphorylase and triacylglycerol lipase and transcripts of the AKHR are most prominently found in fat body tissue [see reviews by (5–7)]. As with most endocrine regulatory peptides, additional functions such as inhibition of anabolic processes (protein and lipid syntheses), involvement in oxidative stress reactions and egg production inter alia are known making AKH a truly pleiotropic hormone [see reviews by (8, 9)]. This is also true for CRZ which is mainly synthesized in neuroendocrine cells of the pars lateralis of the protocerebrum and released via the corpora cardiaca (10). Although originally described as a potent cardiostimulatory peptide (11), it does not generally fulfill this role but is known for many additional functions such as involvement in the (i) release of pre-ecdysis and ecdysis triggering hormones, (ii) reduction of silk spinning rates in the silk moth, (iii) pigmentation events (darkening) of the epidermis in locusts during gregarization and (iv) regulation of caste identity in an ant species (12–15). The functional role of ACP is less clear. All previous studies had not found a clear-cut function for this peptide until work by Zhou and colleagues claimed that ACP in the cricket *Gryllus bimaculatus* regulates the concentration of carbohydrates and lipids in the haemolymph (16).

Experiments in the current study have been conducted with the mosquito species *Aedes aegypti* for a number of reasons. Firstly, *A. aegypti* is an infamous disease vector for pathogens such as Yellow and Dengue fever, Chikungunya and, as latest addition, Zika arboviruses; summarily these are responsible for affecting approximately 50–100 million people per year (17–19). Secondly, knowledge on the interaction of the ligands with their respective receptor are thought to be very helpful for drug research using specific GPCRs as targets for development of selective biorational insecticides (20–22). Another reason was that each of the three neuropeptide systems has already been partially investigated, thus, this study could build on previous research data. With respect to the peptides, these were first predicted from the genomic work on *A. aegypti* (23). The presence of mature AKH and corazonin, but not of ACP, was shown by direct mass profiling (24). The AKH and ACP precursors have been cloned (25) as have one CRZ receptor (CRZR) and multiple transcript variants of the AKHR and ACPR (25–27). Each receptor is very selective and responds only to its cognate peptide, thus there are indeed three completely separate and independently working neuroendocrine systems active in this mosquito species. Here, we hone in on two of these systems, specifically ACP/ACPR and AKH/AKHR, which share the closest evolutionary relationship (2, 28–30), and examined structural features of each ligand important for conferring receptor specificity and activation.

With respect to the ACP system in *A. aegypti*, expression studies revealed transcripts of the major ACPR form as well as the ACP precursor were enriched during development in adults, specifically during day one and four after eclosion (25, 27). On the tissue level, ACP precursor transcripts are mainly present in the head/thorax region (25) and, more specifically, within the brain and thoracic ganglia (27) and the ACPR transcripts are mainly detected in the central nervous system with significant enrichment in the abdominal ganglia, particularly in males (27). The AKH precursor transcripts are present in head/thorax region of pupae and adult females and in the abdomen of males (25). Expression of AKHR was shown in all life stages (25), but similar to the ACPR and ACP transcripts, AKHR transcript variants were found to be significantly enriched in early adult stages (26). On the tissue level, highest expression in adults was found in thorax and abdomen with low levels detected in ovaries (25). Similar expression profiles were determined recently by Oryan et al. (26) using RT-qPCR: expression in head, thoracic ganglia, accessory reproductive tissues, and carcass of adult females as well as in abdominal ganglia and specific enrichment in the carcass and reproductive organs of adult males.

Thus, although quite a substantial body of information on the three signaling systems in *A. aegypti* is known, we lack knowledge of how the various ligands interact with their cognate receptor. For instance, which amino acid residue in the ligand is specifically important for this interaction? By replacing each residue successively with simple amino acids such as glycine or alanine one can probe into the importance of amino acid side chains and their characteristics in relation to facilitating receptor specificity. Such structure-activity studies have been conducted on the AKH system in a number of insects and a crustacean either by measuring physiological actions *in vivo* (31, 32) or in a cellular mammalian expression system *in vitro* (33–35). In conjunction with nuclear magnetic resonance (NMR) data on the secondary structure of AKHs (36, 37) and the knowledge of the receptor sequence, molecular dynamic methods can devise models how the ligand is interacting with its receptor (38, 39). Such a comprehensive study carried out recently on the locust AKH receptor demonstrated that all three endogenous AKH peptides, including two octa- and one decapeptide, interact with similar residues and share the same binding site on their receptor (40).

Since no such data is available for any ACP system, the objective of the current study was to fill this knowledge gap by determining critical amino acids of the ACP neuropeptide necessary for activation of its cognate receptor, ACPR. We therefore designed a series of analogs based on the endogenous *A. aegypti* ACP sequence and screened them using an *in vitro* receptor assay system to determine the crucial residues for ACPR activation. Given the closer evolutionary relationship between the ACP and AKH systems in arthropods (2, 28–30), a second aim of the current study was to advance our knowledge and understanding of critical residues and properties of uniquely positioned amino acids necessary for the specificity of the AKH ligand to its receptor, AKHR-IA. Given the extensive earlier studies examining AKH structure activity relationships, which established that aromatics at positions 4 and 8 and blocked N- and C-terminal ends are critical, we took advantage of naturally occurring AKHs from other insects to examine other positions with amino acid substitutions and determine the consequences onto AKHR-IA activation using the same *in vitro* heterologous assay. Collectively, determining indispensable amino acid residues of these two GnRH-related mosquito neuropeptides that are necessary for activation of their prospective receptors will help clarify how these evolutionarily-related systems uphold specific signaling networks avoiding cross activation onto the structurally related, but functionally distinct signaling system.

## Materials and Methods

### ACP and AKH Receptor Expression Construct Preparation

A mammalian expression construct containing the ACPR-I ORF (hereafter denoted as ACPR) with Kozak translation initiation sequence had been previously prepared in pcDNA3.1^+^ (27); however, this earlier study revealed that coupling of the ACPR with calcium mobilization in the heterologous expression system was not very strong, even following application of high concentrations of the native ACP ligand. Therefore, to improve the bioluminescent signal linked to calcium mobilization following receptor activation, which was required in order to provide greater signal to noise ratio when testing ACP analogs with substitutions leading to intermediate levels of activation of the ACPR, we created a new mammalian expression construct using the dual promoter vector, pBudCE4.1. Specifically, we utilized a cloning strategy as reported previously (41) whereby the ACPR was inserted into the multiple cloning site (MCS) downstream of the CMV promoter in pBudCE4.1 using the sense primer *Sal*I-KozakACPR, 5′-GGTCGACGCCACCATGTATCTTTCGG-3′ and anti-sense primer *Xba*I-ACPR, 5′-TCTAGATTATCATCGCCAGCCACC-3′, which directionally inserted the ACPR within the MCS downstream of the CMV promoter. Secondly, the murine homolog (Gα15) of the human promiscuous G protein, Gα16, which indiscriminately couples a wide variety of GPCRs to calcium signaling (42) was inserted into the MCS downstream of the EF1-α promoter in pBudCE4.1 using the sense primer *Not*I-Gα15, 5′-GCGGCCGCCACCATGGCCCGGTCCCTGAC-3′ and anti-sense primer *Bgl*II-Gα15, 5′-AGATCTTCACAGCAGGTTGATCTCGTCCAG-3′, which directionally inserted the murine Gα15 within the MCS downstream of the EF1-α promoter.

The AKHR mammalian expression construct used in the current study, specifically AKHR-IA which was the receptor isoform that exhibited the greatest sensitivity to its native AKH ligand, was prepared previously in pcDNA3.1^+^ (26) and coupled strongly with calcium signaling in the heterologous system so there was no need utilize the dual promoter vector containing the promiscuous Gα15 as was necessary for ACPR. For each construct, an overnight liquid culture of clonal recombinant bacteria containing the appropriate plasmid construct was grown in antibiotic-containing LB media and used to isolate midiprep DNA using the PureLink Midiprep kit (Life Technologies, Burlington, ON). Midiprep samples were then quantified and sent for sequencing as described previously (26, 27).

### Cell Culture, Transfections, and Calcium Bioluminescence Reporter Assay

Functional activation of the AedaeACPR and AedaeAKHR-IA receptors was carried out following a previously described mammalian cell culture system involving a Chinese hamster ovary (CHO)-K1 cell line stably expressing aequorin (43). Cells were grown in DMEM:F12 media containing 10% heat-inactivated fetal bovine serum (Wisent, St. Bruno, QC), 200 μg/mL Geneticin, 1x antimycotic-antibiotic to approximately 90% confluency, and were transiently transfected using either Lipofectamine LTX with Plus Reagent or Lipofectamine 3000 transfection systems (Invitrogen, Burlington, ON) following manufacturer recommended DNA to transfection reagent ratios. Cells were detached from the culture flasks at 48 h post-transfection using 5 mM EDTA in Dulbecco's PBS. Cells were then prepared for the receptor functional assay following a procedure described previously (27). Stocks of peptide analogs of ACP and AKH were used for preparation of serial dilutions all of which were prepared in assay media (0.1% BSA in DMEM:F12) and loaded in quadruplicates into 96-well white luminescence plates (Greiner Bio-One, Germany). Cells were loaded with an automatic injector into each well of the plate containing different peptide analogs at various concentrations as well as negative control (assay media alone) and positive control wells (50 μM ATP). Immediately after injection of the cells, luminescence was measured for 20 s using a Synergy 2 Multi-Mode Microplate Reader (BioTek, Winooski, VT, USA). Calculations, including determination of EC_50_ values, were conducted in GraphPad Prism 7.02 (GraphPad Software, San Diego, USA) from dose-response curves from 3 to 4 independent biological replicates.

### Synthetic Peptides and Analogs

Peptide analogs based on the native ACP sequence were designed and synthesized by Pepmic Co., Ltd. (Suzhou, China) at a purity of over 90%. All synthetic peptides were initially prepared as stock solutions at a concentration of 1 mM in dimethyl sulfoxide. All stocks of arthropod AKH analogs were prepared similarly to the ACP synthetic peptide analogs and were commercially synthesized by either Pepmic Co., Ltd. (Suzhou, China) or by Synpeptide Co. (Shanghai, China) at a purity of over 90% verified by HPLC and mass spectrometry. Specific sequence information for each of the peptides (and analogs) used on the ACP and AKH receptors examined in this study are provided in Tables 1, 2, respectively.

**Table 1:** Primary structure of the synthetic ACP analogs designed, a natural ACP analog along with extended AKH decapeptides that were tested on the *A. aegypti* ACP receptor (AedaeACPR).

**Peptide/Analog**	**Primary structure of analog**	**Activity on *A. aegypti* ACPR (EC_**50**_)**	**Fold-reduction in activity relative to AedaeACP**
Aedae-ACP	pQVTFSRDWNAamide	2.394E−08	–
Aedae-ACP (A1)	**A**VTFSRDWNAamide	1.576E−06	411.1
Aedae-ACP (A2)	pQ**A**TFSRDWNAamide	7.052E−07	69.8
Aedae-ACP (A3)	pQV**A**FSRDWNAamide	2.26E−05	550.8
Aedae-ACP (A4)	pQVT**A**SRDWNAamide	ND	>1,000
Aedae-ACP (A5)	pQVTF**A**RDWNAamide	1.038E-06	44.9
Aedae-ACP (A6)	pQVTFS**A**DWNAamide	6.872E−07	23.3
Aedae-ACP (A7)	pQVTFSR**A**WNAamide	3.863E−07	15.9
Aedae-ACP (A8)	pQVTFSRD**A**NAamide	ND	>1,000
Aedae-ACP (A9)	pQVTFSRDW**A**Aamide	4.96E−09	0.2
Aedae-ACP (-OH)	pQVTFSRDWNA**-OH**	ND	>1,000
Aedae-ACP (A1b)	**Acetyl-A**VTFSRDWNAamide	5.272E−07	43.6
Aedae-ACP (1-9)	pQVTFSRDWN-amide	4.153E−06	434.2
Aedae-ACP (1-8)	pQVTFSRDW–amide	2.156E−06	410.1
Aedae-ACP (1,3-10)	pQ-TFSRDWNAamide	ND	>1,000
Aedae-ACP (+G)	pQVTFSRDWNA**G**amide	1.113E−07	5.6
Grybi-ACP	pQ**I**TFSRDWNAamide	3.255E−07	13.6
Bommo-AKH	pQLTFTPGWGQamide	ND	>1,000
Lacol-AKH	pQLTFTSSWGGamide	ND	>1,000
Helze-AKH	pQLTFSSGWGNamide	ND	>1,000

*Using heterologous expression of ACPR, the half maximal effective concentration for each of the analogs was determined as well as the corresponding reduction in activity, highlighting how a particular residue substitution or other modification is tolerated in this system. ND = represents no determined EC_50_ value due to minimal or no detectable activity of the analog when tested up to 10 μM*.

**Table 2:** Primary structure of select naturally occurring AKH analogs from insects and their activity on the *A. aegypti* AKHR-IA receptor (AKHR-IA).

**Peptide/Analog**	**Species origin**	**Primary structure of analog**	**Activity on *A. aegypti* AKHR-IA (EC_**50**_)**	**Fold-reduction in activity relative to Aedae-AKH**
Aedae-AKH	*Aedes aegypti*	pQLTFTPSWamide	2.159E−09	–
Lacol-AKH-7mer	^*^Laconobia oleracea	pQLTFT**S**S**-**amide	ND	>10,000
Grybi-AKH	*Gryllus bimaculatus*	pQ**VN**F**STG**Wamide	2.23E−06	1031.5
Libau-AKH	*Libellula auripennis*	pQ**VN**FTPSWamide	1.18E−06	547.9
Pyrap-AKH	*Pyrrhocoris apterus*	pQL**N**FTP**N**Wamide	7.269E−07	336.7
Erysi-AKH	*Erythemis simplicicollis*	pQL**N**FTPSWamide	6.69E−07	309.9
V^2^-PeramCAH-II	^*^Periplaneta americana	pQ**V**TFTP**N**Wamide	1.485E−07	68.8
Bommo-AKH	*Bombyx mori*	pQLTFTP**G**W**GQ**amide	2.362E−08	10.9
Hipes-AKH-I	*Hippotion eson*	pQLTFT**S**SWamide	1.647E−08	7.6
Peram-CAH-II	*Periplaneta americana*	pQLTFTP**N**Wamide	2.226E−09	1.0
Tabat-AKH	*Tabanus atratus*	pQLTFTP**G**Wamide	1.522E−09	0.7

*Using heterologous expression of AKHR-IA, the half maximal effective concentration for each of the analogs was determined as well as the corresponding reduction in activity, highlighting how a particular single residue substitution, a combination of substitutions or other modifications are tolerated in this system. ND = represents no determined EC_50_ value due to minimal or no detectable activity of the analog when tested up to 10 μM*.

* *Denotes a synthetic analog that is based on an endogenous peptide from this insect*.

## Results

### ACP Synthetic Analog Activity on Mosquito ACPR

**Figure 1:**
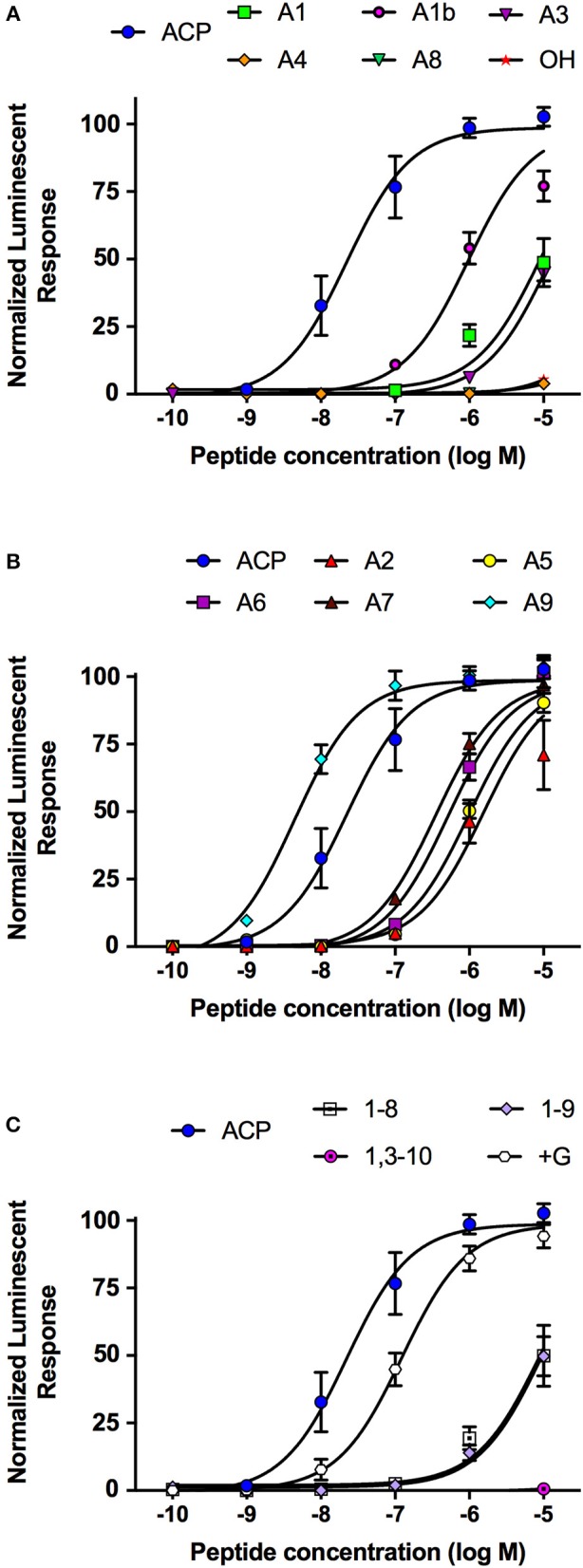
Dose–response curves of the native *A. aegypti* ACP along with synthetic analogs that contain single substitutions or other modifications, which were tested for their activity on the ACP receptor (ACPR) using a cell-based bioluminescent assay. **(A)** Single amino acid substitutions with alanine or modifications to the normally blocked N- or C-termini resulting in a significant reduction to their activity on the ACPR. **(B)** Single amino acid substitutions with alanine to the native ACP sequence having minimal effects to the activity of the tested analogs indicating these substitutions are well tolerated. **(C)** Analogs with internal or terminal truncations or extension (i.e., +Gly analog) relative to the native ACP sequence conferring significant reductions to their activity on the ACPR. The half maximal effective concentration (EC_50_) for each analog along with the corresponding change in activity relative to native *A. aegypti* ACP is provided in Table 1. Luminescence is plotted relative to the maximal response achieved when 10^−5^M Aedae-ACP was applied to the ACPR. Data represent mean +/− standard error of three independent biological replicates.

**Figure 2:**
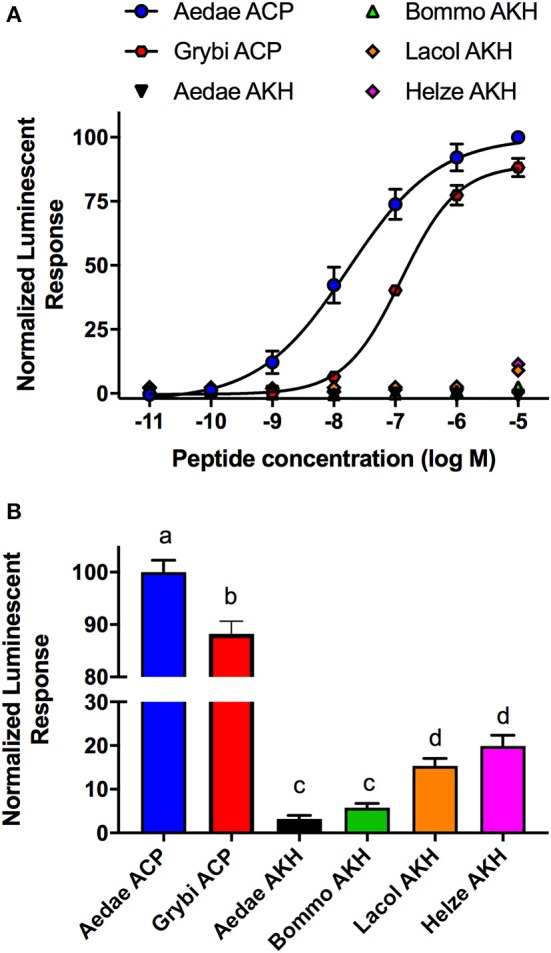
Activity of native ACP analogs from *A. aegypti* and *G. bimaculatus* along with selected naturally-occurring AKH decapetides tested for their activity on the ACP receptor (ACPR) using a cell-based bioluminescent assay. **(A)** Dose–response curves of natural ACP analogs and selected insect AKH decapeptides on the *A. aegypti* ACPR. **(B)** Analysis of the mosquito ACP receptor activation at the highest tested dose (10 μM) indicating activity of Grybi-ACP is significantly reduced as a result of the single substitution at position two. The native Aedae-AKH along with Bommo-AKH were inactive on *A. aegpyti* ACPR while Lacol-AKH and Helze-AKH showed low activity. The half maximal effective concentration (EC_50_) for each analog along with the corresponding change in activity relative to native *A. aegypti* ACP is provided in Table 1. Luminescence is plotted relative to the maximal response achieved when 10^−5^M Aedae-ACP was applied to the ACPR. Data represent mean ± standard error of three independent biological replicates. In **(B)**, different letters denote significant difference (*p* < 0.01) as determined by ANOVA and Tukey post-test.

Analogs of native ACP with single alanine substitutions at each amino acid were synthesized and evaluated by examining their functional activation of the *A. aegypti* ACP receptor (ACPR) expressed using a heterologous system. Carboxy-terminal amidation of ACP was found to be critical for bioactivity as were the two aromatic residues of ACP, including the phenylalanine in the fourth position or tryptophan in the eighth position, since analogs modifying these key features demonstrated no activation of ACPR up to the highest tested concentration of 10 μM (Figure 1A, Table 1). Analysis of single residue alanine substitutions revealed that the next most critical residues for retaining ACPR functional activation were the polar threonine in position three and the blocked pyroglutamate (pGlu) at the amino terminus, which upon replacement with alanine resulted in a ~551- and ~411-fold reduced activity, respectively, compared to native ACP (Figure 1A, Table 1). Alternative blocking of the amino terminus, using N-Acetyl-alanine instead of pGlu, revealed this analog was 10-times more active than the non-blocked one, having only ~44-fold reduced activity compared to native ACP (Figure 1A). Substitutions with analogs containing alanine at the second (valine), fifth (serine), sixth (arginine), and seventh (aspartic acid) positions, had noticeably less impact on the reduction of activity (Figure 1B), by ~70, ~45, ~23, and ~16-fold, respectively. The analog containing an alanine substitution for the asparagine in position nine resulted in a more potent ACPR activation, leading to a ~5-fold greater activity compared to the native ACP peptide (Figure 1B). C-terminally truncated analogs, which lacked either the alanine residue in the tenth position alone or both asparagine and alanine in positions nine and ten, were almost completely inactive, demonstrating ~434 and ~410-fold reduced ACPR activation compared to native ACP (Figure 1C). A C-terminally extended analog, an undecapeptide (i.e., polypeptide with 11 amino acids residues), ending with a glycine amide on the C-terminus elicited a small decrease of activity by about ~6-fold (Figure 1C). The internally truncated ACP analog lacking a valine in the second position also was without any effect, similar to the two aromatic residue substituted analogs or the non-amidated ACP analog (Figure 1C). An ACP analog from the cricket *Gryllus bimaculatus* (Grybi-ACP) was used to further examine the importance of the second position valine in Aedae-ACP, which is replaced by an isoleucine in *G. bimaculatus*. Grybi-ACP was quite active compared to Aedae-ACP but nonetheless demonstrated nearly ~14-fold reduced activity and failed to reach full ACPR activation even when the highest concentration was tested (Figure 2A). As shown previously, the endogenous octapeptide Aedae-AKH did not activate the ACPR at all; however, a subset of the three naturally-occurring insect AKH decapeptides tested here caused some activation, albeit very weak with Lacol-AKH and Helze-AKH leading to between 15 and 20% ACPR activation at the highest tested concentrations (Figure 2B).

### Activity of Natural Arthropod AKH Analogs on Mosquito AKHR-IA

**Figure 3:**
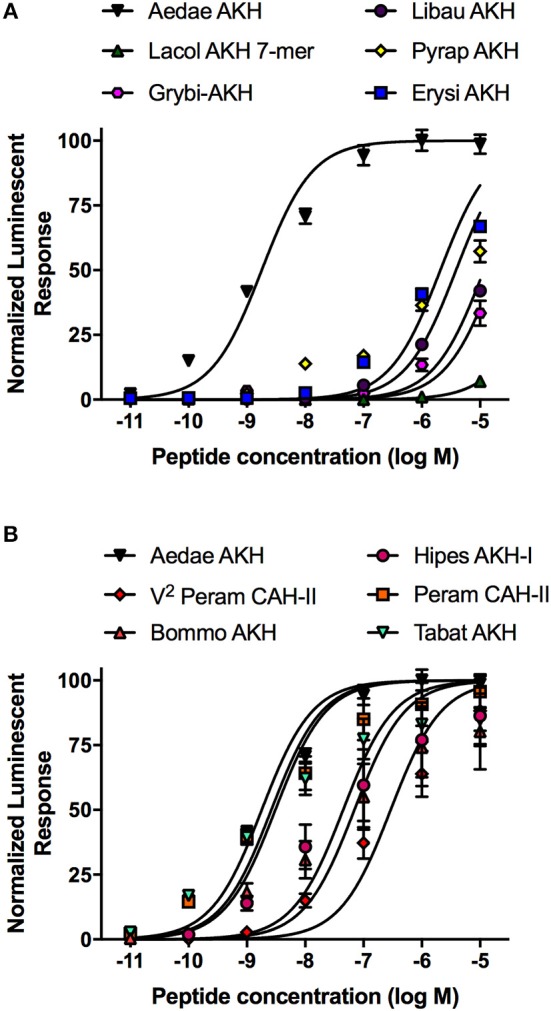
Dose–response curves of the native *A. aegypti* AKH along with mostly naturally occurring analogs from a variety of insects that contain substitutions or other modifications, which were tested for their activity on the AKHR-IA receptor using a cell-based bioluminescent assay. **(A)** Naturally occurring insect AKH analogs having critical substitutions that are not well tolerated by the AKHR-IA, with moderately compromised activity relative to the native *A. aegypti* AKH. **(B)** Naturally occurring insect AKH analogs, or a synthetic analog (V^2^-Peram-CAH-II), having substitutions that are well tolerated by the AKHR-IA, with marginally compromised or slightly improved activity relative to the native *A. aegypti* AKH. The half maximal effective concentration (EC_50_) for each analog along with the corresponding change in activity relative to native *A. aegypti* AKH is provided in Table 2. Luminescence is plotted relative to the maximal response achieved when 10^−5^M Aedae-AKH was applied to the AKHR-IA. Data represent mean ± standard error of three independent biological replicates.

Taking advantage of the variety of natural AKH analogs in arthropods, we examined a subset of insect AKH peptides (see Table 2) with unique substitutions compared to the endogenous mosquito AKH peptide and determined their activity on the AKH receptor, *A. aegypti* AKHR-IA. From this analysis, the least effective analog tested was the C-terminally truncated peptide, Lacol-AKH-7mer, with only ~7% AKHR-IA activation at the highest tested concentration (10 μM), which represents a reduced efficacy of at least five orders of magnitude (Figure 3A). The next least active AKH analog was the cricket peptide, Grybi-AKH, which has five residues substituted over the length of the octapeptide compared to Aedae-AKH (see Table 2), and the activity of this analog on mosquito AKHR-IA is compromised by over three orders of magnitude (1,032-fold reduced activity) as a result of these combined substitutions (Table 2 and Figure 3A). The AKH from the dragonfly, *Libellula auripennis* (Libau-AKH), with substitutions at positions two and three only, had a nearly 2-fold improved activation over that achieved by Grybi-AKH; however, the activity of this peptide on the *A. aegypti* AKHR-IA was still reduced by 548-fold compared to Aedae-AKH (Table 2). The AKH from the firebug *Pyrrhocoris apterus* (Pyrap-AKH) that has two substitutions in positions three and seven, both asparagine, is better tolerated than Libau-AKH and Grybi-AKH but is still less active on the *A. aegypti* AKHR-IA being compromised by 337-fold relative to Aedae-AKH (Figure 3A). To better understand the importance of N-terminal residues, we tested an AKH analog from the dragonfly *Erythemis simplicicollis* (Erysi-AKH), which is only different to Aedae-AKH at position three (threonine to asparagine; see Table 2); Erysi-AKH had 310-fold less activity compared to Aedae-AKH (Figure 3A). The peptide named V^2^-Peram-CAH-II, which is a synthetic analog of one of the two endogenous peptides of the American cockroach, *Periplaneta americana*, does not occur in this species and is characterized by a second position substitution of valine for leucine along with the seventh position substitution of asparagine for serine (see Table 2). AKHR-IA activation by V^2^-Peram-CAH-II was found much improved, albeit still 69-fold less effective compared to the native *A. aegypti* AKH (Figure 3B). If the C-terminus is extended by two amino acids as implemented by utilizing the AKH analog from the domestic silkmoth, *Bombyx mori* (Bommo-AKH), having glycine at position nine, glutamine at position 10 and a glycine at position seven (instead of the serine in Aedae-AKH), revealed activation of AKHR-IA was reduced by only 11-fold (Figure 3B). Finally, examining natural analogs of this peptide family containing single substitutions in the C-terminal region, at positions six or seven, it was demonstrated that these changes result in only a slight reduction of activation. The cockroach AKH, Peram-CAH-II (seventh position serine substituted with asparagine) had near identical efficacy to the native mosquito AKH while the AKH analog from the sphingid moth, *Hippotion eson* (Hipes-AKH-I; serine substitution for proline in the sixth position), had only an 8-fold reduced activity (Figure 3B). The AKH analog from the black horse fly, *Tabanus atratus* (Tabat-AKH; glycine substitution for serine at position seven), had an activation that partially exceeded (~30% improved efficacy) the activity of the native mosquito AKH (see Table 2; Figure 3B).

## Discussion

The current study set out to examine for the first time the ligand structure-activity relationship for an insect ACP receptor. This is of utmost importance in order to gain insight on the specific structural features of the ACP system given that extensive studies have been carried out previously on AKH receptors in a variety of species using natural and synthetic analogs of this neuropeptide family (32, 33, 44–57). For comparative reasons, we also studied the AKH receptor of *A. aegypti* but did not examine all residues since a plethora of previous research had clearly identified critical residues, for example the aromatics in positions four and eight (see references above). By examining the activity of a variety of ACP analogs on the activation of the *A. aegytpi* ACP receptor (ACPR), including single alanine substitutions along with truncated or extended analogs, a few observations can be highlighted. Firstly, the overall charge of the peptide does not appear to play a role with regards to its influence on receptor activation since substitution of the basic (arginine) or acidic (aspartic acid) residues did not lead to a highly detrimental effect. Specifically, one would assume that the ACP receptor may prefer a neutral ligand given that the native ACP has no net charge (i.e., is neutral) since it has both a single basic and acidic residue. However, the modified analogs which replace the basic (position 6) or acidic (position 7) residues with alanine, create negatively or positively charged molecules, respectively, with both being quite active and so neither proved to be detrimental substitutions, suggesting that the extra carboxy- or amino group is not involved in forming a salt bridge with the receptor. Secondly, with the exception of the aromatic tryptophan residue in the eighth position, the C-terminal residues of ACP do not appear to be highly important since alanine substitutions in positions five through seven only marginally impacted activity between ~16 to 45-fold whereas alanine substitution in position nine resulted in this analog having 5-fold increased activity. Such a compound may be a good lead substance for the development of a superagonist in future studies, with the eventual goal to design an active non-peptidergic mimetic. Amidation of the C-terminal residue was critical since the free acid analog exhibited no activity; however, it is noteworthy that the C-terminally extended peptide having an amidated glycine was also quite effective with ACPR activation compromised by only 6-fold. Previous studies examining free acid analogs of AKH (including both *in vitro* and *in vivo* assays) have demonstrated that in some cases the absence of a blocked C-terminus is less critical (33, 51, 58), whereas other investigations have revealed the presence of an amidated C-terminus to be very important with poor or no responses with analogs lacking this feature (31, 59). These observed differences in response to key structural elements, including the normally amidated C-terminus of AKH, may reflect the occurrence of inter- and intra-species receptor subtypes which may be differentially sensitive to these modifications (31, 58), and could also point to the well documented phenomenon seen for AKH receptors that are more promiscuous whereas others are very strict in the structural characteristics of their particular ligand (33, 34, 59). This latter scenario is more in-line with observations for the *A. aegypti* ACP system where only a single functional receptor variant occurs (27), which we show herein does not tolerate the absence of the C-terminal amidation and is thus of high importance for the ACP system. Thirdly, both aromatic residues were essential for activity on the ACPR while N-terminal residues of the peptide, including mainly threonine and pyroglutamic acid, were also highly critical since alanine substitutions were not tolerated leading to significantly reduced activity of these analogs relative to the native ACP peptide. However, alternatively blocking this alanine substitution on the N-terminus through acetylation improved the activity of this analog by 10-fold. Similar observations have been made with AKH analogs tested using both *in vivo* and *in vitro* assays whereby complete removal of the N-terminal pyroglutamate abolishes activity of the AKH analog whereas the alternatively blocked N-terminus (i.e., N-Acetyl-Ala), or even simply glutamate or glutamine that can spontaneously undergo cyclization (60), were shown to have improved activity, albeit lower than the native AKH (31, 54, 58). Interestingly, alanine substitution for valine in the second position, thus both hydrophobic but the former having a shorter side chain, led to a relatively minor effect on ACPR activation (~70-fold reduced activity). Comparatively, replacing valine at position two with another aliphatic amino acid with a hydrophobic but longer side chain as in Grybi-ACP (isoleucine at position two) had only a minor effect on ACPR activation with a ~14-fold reduction compared to Aedae-ACP, indicating that side chain length at this position may be important for ligand-receptor interaction. The internally truncated analog omitting valine within the N-terminal region elicited no ACPR activation, indicating that the spacing between the more critical residues, namely pyroglutamate and polar threonine, is essential for ACP peptide activity on the ACPR. Thus, perhaps in common with AKH structural features, the switch between adjacent hydrophobic and hydrophilic residues is necessary in order to properly “fit” and bind with its particular receptor (31) so that not only is the ACP analog shorter by one amino acid (a non-apeptide), but moreover, the entire structural configuration of the peptide is disrupted and the whole sequence is no longer in sync with its binding pocket on the receptor. This corroborates with studies that have examined the conservation of the ACP primary structures across insects from different orders (including Diptera, Lepidoptera, Coleoptera, Hymenoptera and Hemiptera) as well as from various species within the same order (seventeen species of Coleoptera), where the N-terminal pentamer sequence, when the ACP system is present, is nearly completely conserved across these various species with a consensus motif of pQVTFS-, whereas significant sequence variability occurs within the C-terminus of the ACP sequence with deca-, nona- and even dodecapeptides reported (2, 61).

Our studies on the importance of specific amino acids of the ligand Aedae-AKH for activating the mosquito AKH receptor were driven by a number of previous studies on AKH receptors utilizing *in vitro* heterologous assays as well as *in vivo* bioassays monitoring lipid- and/or carbohydrate-mobilizing actions of AKH analogs. For instance, the blocked N- and C-termini were mostly quite important and the aromatics at positions 4 and 8 were absolutely essential (46, 47, 53, 54). Furthermore, it was demonstrated earlier that certain AKH receptors tested *in vitro* were found to be quite promiscuous reacting well to ligands that had many substitutions, for example the AKH receptors from the desert locust *Schistocerca gregaria* and the pea aphid *Acyrthosiphon pisum* (34). On the other hand, a group of AKH receptors from other insects, including the fruit fly *Drosophila melanogaster* and the mosquito *A. aegypti*, were found to be highly specific, tolerating only minor substitutions (33, 34). However, since only a few modifications to the natural AKH ligand were tested on the *A. aegypti* receptor, in the present study we focused on the importance of residues at positions two and three at the amino end and five through seven at the carboxyl end by utilizing naturally-occurring AKH bioanalogues containing all or a subset of these substitutions. Starting with Grybi-AKH, which differs from Aedae-AKH in all the variable positions except the prototypical features of any insect AKH such as pyroglutamic acid at the first position, phenylalanine and tryptophan at positions four and eight, respectively, and an amidated C-terminus, we demonstrated a large reduction in activation of the Aedae-AKHR-IA by over three orders of magnitude. This gave us the opportunity to tease out which of the five positions are the main drivers for this observed low activity. Previous studies using modifications at positions five and seven together had shown that such ligands resulted only in a slight loss of receptor activation (33, 34, 58). In line with this observation, position six was not found to be essential for AKHR-IA activation since a single amino acid change from the uniquely shaped imino acid proline to a serine residue (as found in Hipes-AKH-I), resulted in <10-fold loss in activity on AKHR-IA. Here, we clearly established that position seven, although changed either from a polar side chain in serine to a much larger polar side chain in asparagine (Peram-CAH-II), or to the shortest amino acid glycine (Tabat-AKH), is not essential for activation of AKHR-IA since these analogs had comparable activity to the native Aedae-AKH. In fact, to our surprise, Tabat-AKH may serve as a potential lead substance for design of a superagonist since this natural AKH analog elicited 30% improved activity relative to the native mosquito AKH. It appears that the more flexible and non-polar glycine residue allows the ligand a tighter binding to the receptor than the polar and neutral serine. This is particularly interesting given the permissiveness of substitutions in the vicinity of the C-terminus, which may allow a greater variety of lead compounds to be designed and tested that could interfere with this neuropeptide system and perturb its normal functioning vital for mobilizing energy substrates in insects. Interestingly, Bommo-AKH retained strong activity on the *A. aegypti* AKHR-IA, which in common with Tabat-AKH has a glycine substitution in position seven, but also has an extended C-terminus containing glycine and glutamine following after tryptophan in the eighth position. This indicates that the extended nature of this AKH ligand does not compromise AKHR-IA activation and follows with the earlier observations on the overall permissiveness of the C-terminal region of the AKH ligand.

To corroborate that the substitutions in positions two to three were of greatest importance, we examined the activity of a natural AKH analog from *L. auripennis* (Libau-AKH), whose sequence matches that of native Aedae-AKH except for positions two to three (valine and asparagine) that are instead shared with Grybi-AKH. Indeed, our results confirmed that the substitutions in this N-terminal region are much more critical and are not well tolerated by AKHR-IA in *A. aegypti* since Libau-AKH displayed only a marginal improvement (i.e., nearly 2-fold) compared to Grybi-AKH. Supporting the notion that N-terminal residues are most important for AKHR-IA activation, two additional natural AKH analogs were examined having substitutions in either the third position alone (Erysi-AKH) or both the third and seventh position residue (Pyrap-AKH). In comparison to Aedae-AKH, Erysi-AKH had a similar reduction in activity as Pyrap-AKH indicating that the residue in the third position is essential within the natural AKH ligand since it is established that a seventh position substitution has a negligible effect. In light of this evidence indicating substitutions in positions two and three are not well tolerated by the *A. aegypti* AKHR-IA, we aimed to discern which of these two sites were the absolute most critical. Comparing the activity V^2^-PeramCAH-II with that of Pyrap-AKH demonstrates that the valine substitution for leucine in the second position is far better tolerated compared to the asparagine substitution for threonine in the third position, indicating this latter position is indeed the most critical.

Taken together, these finding corroborate recent analysis of select dipteran AKH analogs where structure-activity analyses showed the least tolerated substitutions were localized to the N-terminal region, proving crucial for activation of *D. melanogaster* and *A. gambiae* AKH receptors, which the authors described was due to this core region of the peptide forming a predicted β -strand necessary for receptor interaction (33). Further, this supports our findings that the C-terminal region of the AKH octapeptide, excluding the aromatic tryptophan in the eighth position, is not as critical for activity. As a putative reason for this, it is argued that the side chains of a subset of these amino acids may be buried in the assumed β-turn formed by the residues in the C-terminal region of AKH neuropeptides (31, 33, 46), which is now supported by molecular modeling and nuclear magnetic resonance spectroscopy studies that have confirmed the β-turn configuration in the AKH secondary structure (36, 37, 62, 63). Indeed, a comprehensive structural analysis of the AKH system in the desert locust, *Scistocerca gregaria*, recently confirmed that all three of the native AKH neuropeptides despite exhibiting differences in amino acid composition and in the length of the peptide (i.e., both octa- and decapeptide analogs), nonetheless exhibit a β-turn structure and interact with the same residues within the single locust AKH receptor (40).

Integrating the novel data from the present study analyzing structural characteristics of the two most recently diverged GnRH-related family members in arthropods, we can infer with some degree of confidence how these ligands act only upon their respective receptors. The ACP sequence in many insect species often contain charged residues in positions six, seven or nine, which in instances where only a single basic or acidic residue occurs, provide an overall charge to the peptide (2, 61). However, our results revealed that the overall charge of the ACP analog did not largely compromise activity since alanine substitutions of these charged residues were quite tolerated by the ACPR. Comparatively, AKH receptors do not like charges associated with their ligands since no basic residues occur and only a few natural AKH family members containing an acidic residue (e.g., aspartic acid at position seven) are known, which have been shown to be not well tolerated by AKH receptors in *L. migratoria* and *P. americana* (47, 64, 65). Another feature differentiating the AKH and ACP peptides in *A. aegypti* is the presence of an alternating pattern of hydrophobic and hydrophilic residues over the entire length of the Aedae-AKH, which is shared with other AKH family members (31), whereas the ACP contains a motif of three hydrophilic residues at its core residues 5–7, which may lead to incompatability with the AKHR-IA. Additionally, clearly the valine substitution in position two is not tolerated well by the *A. aegypti* AKHR-IA, and so this residue which is present in position two of the ACP sequence may also contribute toward the inactivity of this peptide on the AKHR-IA. On the other hand, since the AKH peptide from *B. mori* (a decapeptide) was quite active on the *A. aegypti* AKHR-IA, the longer length of ACP is unlikely to be the cause of its inactivity on the AKHR-IA.

Our findings also set the framework toward understanding why the *A. aegypti* AKH has no activity on the ACP receptor. As we saw with the AKHR-IA, substitution of valine for leucine in position two led to nearly a 70-fold reduction in receptor activity. On the other hand, the removal of valine and its replacement with alanine in the ACP sequence led to a similar 70-fold reduction in activity on the ACPR. In contrast, substitution of valine in position two with another branched-chain amino acid (i.e., isoleucine) found in the Grybi-ACP analog, only marginally reduced activity. Thus, the presence of valine in position two is needed for optimal activity for ACPR whereas its absence in the same position (but on the AKH peptide) is necessary for optimal activation of AKHR-IA. Finally, one clear finding from these studies is that ACP analogs that are too short (nonapeptide or octapeptide), even if they contain all the hallmark features, as demonstrated by the C-terminally truncated analogs for example, fail to activate ACPR. Thus, the length of the ligand is most relevant and the binding pocket of ACPR requires at least a decapeptide leaving the *A. aegypti* AKH, an octapeptide, too short for ACPR binding and activation, while comparatively some decapeptide AKH analogs demonstrated low (albeit significant) levels of activity on ACPR.

## Data Availability Statement

The datasets generated for this study are available on request to the corresponding author (J-PP: paluzzi@yorku.ca).

## Author Contributions

GG and J-PP designed the synthetic analogs and wrote the manuscript. AW performed all the experiments and AW, J-PP, and GG analyzed the data. All authors have contributed toward the revisions and have granted approval of the final manuscript submitted for publication.

### Conflict of Interest

The authors declare that the research was conducted in the absence of any commercial or financial relationships that could be construed as a potential conflict of interest.
